# Evidence of Local Concentration of α-Particles from ^211^At-Labeled Antibodies in Liver Metastasis Tissue

**DOI:** 10.2967/jnumed.118.216853

**Published:** 2019-04

**Authors:** Satoshi Kodaira, Yukie Morokoshi, Huizi Keiko Li, Teruaki Konishi, Mieko Kurano, Sumitaka Hasegawa

**Affiliations:** 1 Radiation Measurement Research Team, National Institute of Radiological Sciences, National Institutes for Quantum and Radiological Science and Technology, Chiba, Japan; 2 Radiation and Cancer Biology Team, National Institute of Radiological Sciences, National Institutes for Quantum and Radiological Science and Technology, Chiba, Japan; and; 3 Regenerative Therapy Research Team, National Institute of Radiological Sciences, National Institutes for Quantum and Radiological Science and Technology, Chiba, Japan

**Keywords:** autoradiography, α-particle, ^211^At radionuclide, dose distribution, microscopy, radioimmunotherapy

## Abstract

We investigated the local concentration of α-particles from ^211^At-labeled trastuzumab antibodies against human epidermal growth factor receptor type 2 antigens in liver metastasis tissue of mice. **Methods:** Mice carrying metastatic cancer in their liver were injected with ^211^At-agent. After 12 h, the liver was removed and sliced, and 2 tissue samples of liver tissues without lesions and one containing metastatic lesions were mounted on the CR-39 plastic nuclear track detector. Microscope images of the tissues on the CR-39 were acquired. After irradiation for 31 h, the tissues were removed from the CR-39. A microscope image of α-particle tracks on the CR-39 was acquired after chemical etching. The positions of each tissue sample and the emitted α-particle tracks were adjusted to the same coordinates. **Results:** The positional distribution of α-particle tracks emitted from ^211^At was consistent within the tissue. The α-particle tracks were mainly allocated in the tumor region of the tissue. The absorbed dose in individual cells segmented by 10-μm intervals was obtained by the spectroscopic analysis of the linear-energy-transfer spectrum. The concentration efficiency—the track density ratio of α-particle tracks in the necrotized tissue, which was the tumor region, to the normal tissue—was found to be 6.0 ± 0.2. In the tumor region, the high–linear-energy-transfer α-particles deposited a large enough dose to cause lethal damage to the cancer cells. **Conclusion:** The total absorbed dose ranged from 1 to 7 Gy with a peak at around 2 Gy, which would correspond to a 2–3 times higher biologically equivalent dose because of the high relative biological effectiveness of the α-particles emitted from ^211^At.

Radioimmunotherapy is more efficient with α-particle emitters than with conventional β-ray emitters because the former provides a high linear-energy transfer (LET) that can induce enough damage to break double-stranded DNA, as well as having a short range in tissue in order to target cancer cells but not the surrounding normal cells (1). Recently, ^223^Ra-chloride–emitting α-particles have been approved as a therapeutic agent for bone metastasis because of their specific characteristics, which are similar to those of calcium concentrating into the bone (2–5). Nonclinical and clinical research studies with the radionuclide ^211^At (half-life, 7.2 h), emitting 5.87-MeV α-particles, are being performed as one promising candidate for radioimmunotherapy (6,7). It is expected that ^211^At-labeled monoclonal antibodies will selectively concentrate in the target cancer cells and cause lethal damage there by the high-LET α-particle irradiation. Preclinical models with ^211^At-labeled monoclonal antibodies were investigated for lymphoma and various solid tumors. For example, ^211^At-labeled MX35 F(ab′)2 was identified as effective for ovarian cancer (8–10), and ^211^At-labeled trastuzumab antibody was identified as effective against human epidermal growth factor receptor type 2 (HER2) antigens for peritoneal metastasis of gastric cancer (11).

Notwithstanding the success of ^211^At in preclinical trials with mice, its physical aspects related to dosimetry should be addressed in order to characterize the treatment effectiveness of ^211^At-labeled monoclonal antibodies and optimize treatment conditions such as injection amount and concentration. Because of the short range of α-particles and the heterogeneous distribution of ^211^At-labeled antibodies in tissues, mean absorbed doses calculated by radioactivities from whole organs or tissues may not accurately correlate with the therapeutic outcomes and may predict unfavorable toxicities in normal tissues. In addition, to assess the correlation between biologic responses and dosimetry, it is critically important to evaluate the LET of a single α-particle emitted from ^211^At and its spatial distribution in tissue.

A new digital autoradiography technique called the α-camera has emerged to evaluate the distribution of α-particles in tissue (12), and a spatial resolution of about 35 μm has been achieved for imaging their distribution in tissue. However, the α-camera has limitations in identifying the local concentration of α-particles at a single-cell level (∼10 μm) and in determining the LET of an individual α-particle, and there is contamination due to photons emitted via electron capture and α-decay as well.

An aluminum oxide crystal–based device that detects a nuclear track by imaging a fluorescent track signal with a confocal microscope has been developed for radiation dosimetry (13–15) and single-cell radiobiology applications (16–18). This technology allows for microscopic dosimetry at a single-cell level. However, the available imaging area is strongly limited by the current low scan speed, which is on the order of several days for 1 cm^2^. Therefore, this detector is not applicable to autoradiographic studies of animal samples that are on a scale of centimeters. Thus, no reliable methods have yet been established to assess the LET distribution of α-particles heterogeneously distributed in tissue on a microscopic level.

Recently, we reported that the binding efficiency of ^211^At-labeled antibodies to targeted cells had been verified by a one-to-one correspondence investigation (i.e., the number of α-particle traversals per individual cell) using CR-39 plastic nuclear track detectors (19). The CR-39 is a promising tool for microscopic dosimetry on a single-cell level based on LET spectroscopy with a submicron spatial resolution (20). The high-speed imaging capability of the microscope (21)—currently reaching 30 s/cm^2^—allows autoradiographic imaging of the spatial dose distribution in tissue at a microscopic resolution (19). Moreover, the CR-39 responds only to heavily charged particles (LET ≥ 3.5 keV/μm) without any contamination by photons (20).

In the present study, we used the CR-39 to image the α-particle distribution from ^211^At-trastuzumab antibodies targeting HER2 proteins in mouse liver metastases and to investigate the concentration efficiency and the given dose in the targeted tumors.

## MATERIALS AND METHODS

### α-Particle Detection and Dosimetry

The damage trail left in the CR-39 by the incidence of an α-particle is observable under an optical microscope as a conical etch pit after chemical etching (22). The detector response (*S*) is defined as the etch velocity, which is obtained from the geometric parameters of the etch pit as…

Eq. 1 $$S\equiv \frac{V_{t}}{V_{b}}-1=\sqrt{\frac{16B^{2}D_{A}^{2}}{{\left(4B^{2}-D_{B}^{2}\right)}^{2}}+1}-1.$$

Here, *D*_A_ and *D*_B_ are the major and minor axes of the elliptic etch pit aperture, respectively, and *B* is the removal thickness due to the chemical etching. *S* is scaled as the linear energy transfer (*L*), which is calibrated with various heavy ion beams covering the wide LET range between 3.5 and 600 keV/μm in water (20). The fluence (φ) (cm^−2^) in each LET bin (Δ*L*) yields the LET spectrum. The detectable incident angle in CR-39 is limited by *S* because of the critical angular dependency (23). In other words, a particle with incident angle between π/2 and π/2 – θ_c_ is observable, where θ_c_ is the critical incident angle. The detection efficiency, η (track per particle), is calculated by…

Eq. 2 $$\eta =\frac{\int_{\theta_{c}}^{\pi /2}\sin\theta \cos\theta d\theta }{\int_{0}^{\pi /2}\sin\theta \cos\theta d\theta }=1-\sin^{2}\theta_{c}=\frac{{\left(S+1\right)}^{2}-1}{{\left(S+1\right)}^{2}}.$$

The fluence (φ) is obtained by taking the detection efficiency and angular LET dependency (20,24). The absorbed dose (*D*) is obtained by…

Eq. 3 $$D\left(\text{Gy}\right)=\frac{1.6\times 10^{-9}}{\rho }\underset{i}{\sum }\varphi_{i}L_{i},$$

where ρ is a specific gravity.

We used CR-39 plates (TechnoTrak; Chiyoda Technol Corp.) with dimensions of 25 × 75 × 0.9 mm. The CR-39 plates were used as microscope slides on which sliced tissues were mounted. The plates were also used as the detector of α-particles emitted from the tissues.

### ^211^At-Labeled Monoclonal Antibodies

We produced ^211^At-labeled monoclonal antibodies as described previously (11). Briefly, trastuzumab is a humanized anti-HER2 monoclonal antibody that was purchased from Chugai Pharmaceutical Co. ^211^At was produced using the National Institute of Radiological Sciences AVF-930 cyclotron (25). The ^211^At-labeled trastuzumab (26) was isolated in phosphate-buffered saline with size-exclusion chromatography using a Sephadex 50 spin column (GE Healthcare) at room temperature (centrifuge; 730*g,* 2 min).

### Animal Experiments

All animal experiments were approved by the Animal Care and Use Committee of the National Institute of Radiological Sciences at the National Institutes for Quantum and Radiological Science and Technology and were undertaken in compliance with the institutional guidelines on animal care and handling.

### Biologic Samples

We produced a mouse model of liver metastasis of gastric cancer by injection of 2 × 10^6^ N87/luc cells, which are human gastric cancer NCI-N87 cells stably expressing the luciferase gene (11), into the splenic vein in severe combined immunodeficiency mice (C. B17/lcr-scid/scidJcl (homo); CLEA Japan). We implanted the cells a week before the experiment and confirmed the tumor formation in the liver by in vivo luminescence imaging (Vilber Lourmat). N87/luc cells intrinsically overexpress HER2 (11). ^211^At-trastuzumab (1 MBq) was injected into the tail vein of the mice.

### Experimental

The liver, containing the cancer metastasis, was extracted from the mice 12 h after antibody injection and then sliced at an 8-μm thickness using a freezing microtome (Leica CM 1850; Leica Biosystems). Two sliced tissue samples, one containing the specifically localized tumor and liver tissues without visible metastatic lesions, were mounted on the CR-39 plate. The images of tissues were acquired with a wide-area optical microscope equipped with a ×20, NA0.45 objective lens (FSP-1000; Seiko Time Systems Inc.). The image size was approximately 12 × 12 mm with a pixel size of 0.28 μm/pixel. After 31 h, corresponding to approximately 4 half-life durations of ^211^At decay, the tissues were removed from the CR-39 plate using a 1% solution of polysorbate 20. The CR-39 plate was then etched in 7 M NaOH at 70°C for 2 h. The microscope images of α-particle tracks formed on the CR-39 plate surface were acquired under the same conditions as mentioned above.

The images recording α-particle tracks were analyzed with PitFit software developed for analyzing etched pits in the -39 (21). The position (*x, y*) on the CR-39 coordinates and the α-particle track size (*D*_A_ and *D*_B_, the major and minor axes of the elliptic etch pit) were analyzed. The thickness (*B*) removed by the etching was determined to be 3.3 ± 0.7 μm by measuring the thickness change of the detector with a micrometer (MDC-25 M; Mitsutoyo) before and after etching (27). Then, the detector response (*S*) for each track was obtained from Equation 1 and the individual LET was determined using the calibration function (20).

The coordinates on the CR-39 plate before and after etching were adjusted and corrected by the affine transformation technique based on the locations of 4 markers scratched with a small diamond pen onto surrounding tissues before the experiments (19). The distribution of α-particle tracks was superposed to the location of the sliced livers. The number of tracks located in the region of interest in the tissue sample, especially 2 kinds of color regions (pink and purple), was counted in binarized images as shown in Supplemental Figure 1 (supplemental materials are available at http://jnm.snmjournals.org). For discriminating the blank region from the tissue, the tissue imaged was simply binarized by setting a threshold level (138/256) in 8-bit gray scale. The pink region was extracted by setting band-pass thresholds of 24-bit color levels (red, 123–177; green, 42–84; blue, 94–139).

## RESULTS

**FIGURE 1.**
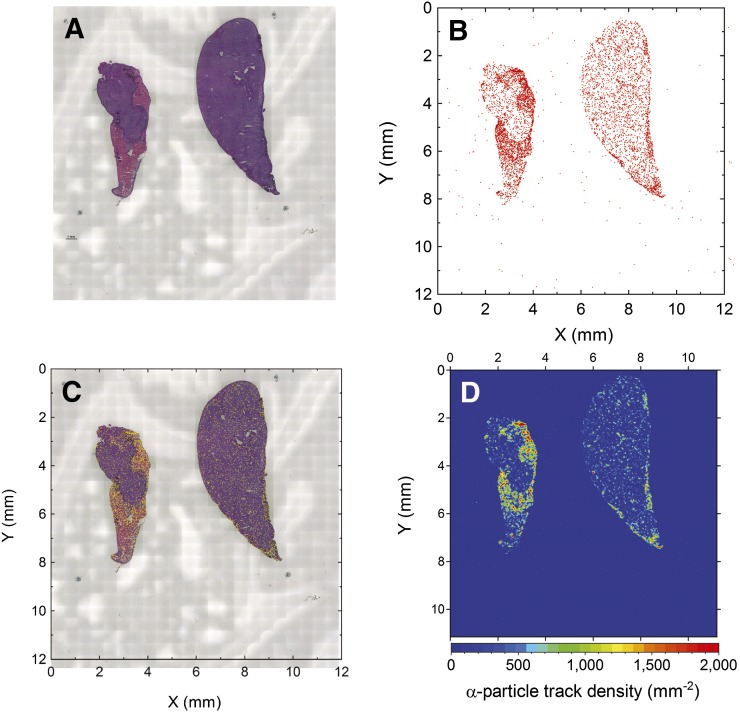
(A) Photomicrograph of sliced tissues; left tissue has specifically localized tumors. (B) Scatterplot of detected α-particle tracks on CR-39. (C) Superposition of tissue image and scatterplot of α-particle tracks. (D) Contour map of α-particle track density in binned positions (Δ*x,* Δ*y*) with 50-μm intervals.

Along the tissues reconstructed from the tiled microscope images (Fig. 1A), we could observe the α-particle tracks emitted from ^211^At distributed in the tissues in the scatterplot of the positions of the individual tracks (Fig. 1B). The superposition of the tissue image and scatter plot of the α-particle tracks is shown in Figure 1C, in which yellow dots correspond to the individual track positions. The intensity map of the α-particle tracks is illustrated in Figure 1D and was visualized by counting the number of tracks in binned positions (Δ*x,* Δ*y*) at 50-μm intervals. The distribution of α-particle tracks was significantly higher in the pink region in the tissue than in the purple region.

**FIGURE 2.**
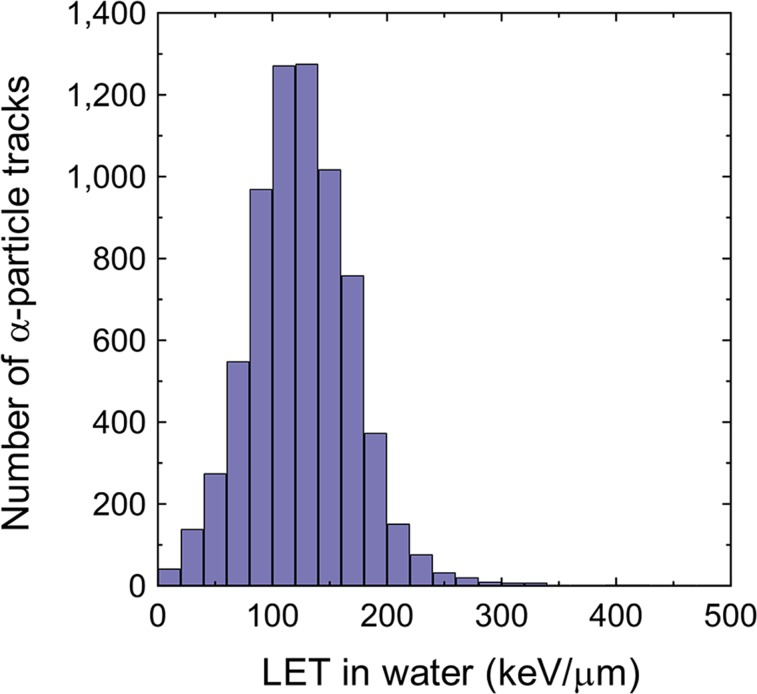
LET spectrum of observed α-particles.

**FIGURE 3.**
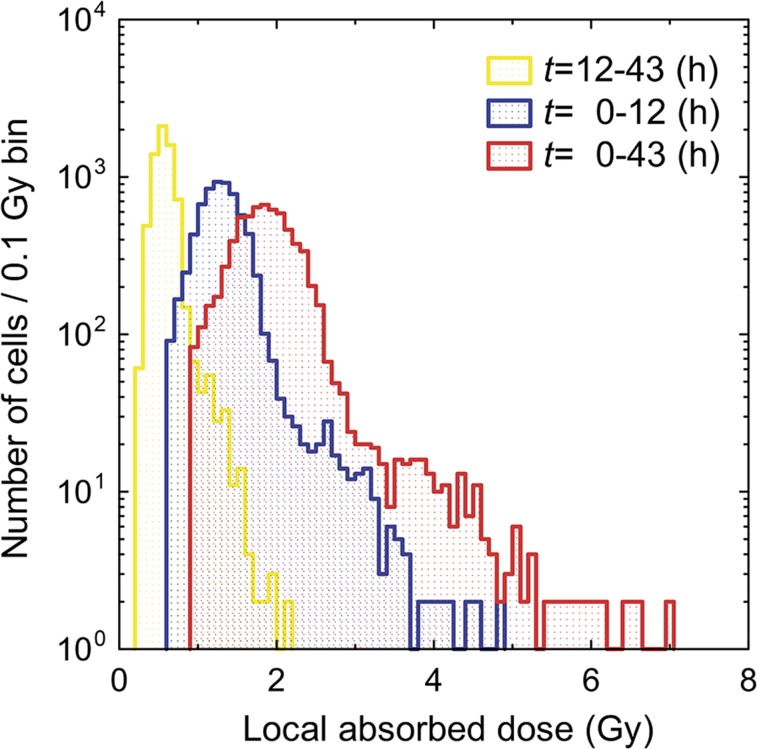
Histogram of observed local absorbed dose for *t* = 12–43 h, estimated dose corrected by half-life of ^211^At decay for *t* = 0–12 h, and estimated total dose for *t* = 0–43 h. Doses are given in cell scale size (10 μm).

LET dosimetry of individual α-particle tracks was performed to assess the absorbed dose in tissues. Figure 2 shows the LET spectrum of observed α-particle tracks with a peak at around 130 keV/μm, as is consistent with previous work using ^211^At-binding cells (19). The absorbed dose (*D*) was calculated using Equation 3 in locally segmented cells binned in a square size of 10 × 10 μm, which is comparable to the typical mean cell diameter of 10 μm (28). Thus, the obtained observed local absorbed dose distribution is shown in the yellow histogram of Figure 3. These data were recorded on the CR-39 for 31 h after the slice-and-mount preparation, meaning that the number of emitted α-particles should be corrected by the reduction rate because of the half-life of ^211^At decay for the 12-h period between antibody injection into the mouse tail vein and the slice-and-mount preparation on CR-39.

## DISCUSSION

### Concentration Efficiency

The α-particle tracks were significantly concentrated in the pink region of the tissue, in contrast to the purple region. The pink region was stained by eosin and thus would be necrotized. We think that the pink region was specifically occupied by tumors, where ^211^At-trastuzumab should be concentrated, causing lethal damage by α-particle irradiation. The track densities were 1,060.9 ± 20.7 mm^−2^ in the pink region and 177.4 ± 6.1 mm^−2^ in the purple region. The concentration efficiency, the track density ratio of pink to purple regions, was 6.0 ± 0.2. Here, the error (1σ) comes from the statistics of observed α-particle track counts. The previous in vivo experiment (11) proved that the ^211^At-trastuzumab concentrated in the tumor (i.e., HER2 distribution) correlates to the double-strand break distribution by the γ-H2AX observation, indicating that the ^211^At-trastuzumab concentration causes the lethal damage to cancer cells. The dose distribution of α-particle tracks correlating to the distribution of necrotized tissue should correlate with the DNA-damaged region in the same experimental system (11). The significant concentration of α-particles in the tumors validates the effectiveness of the used ^211^At-trastuzumab, supporting the in vivo results (11).

### Total Local Absorbed Dose

The assessment of absorbed dose, which is given by the number of α-particles and their LET, is critical to the discussion of lethal damage in cancer cells. The observed distribution of local dose was due to the recorded α-particle tracks on the CR-39 during the time between the slice-and-mount preparation (*t*_1_ = 12 h) and removal of tissue from the detector (*t*_2_ = 43 h) as shown in the yellow histogram of Figure 3. This means that the α-particle emission after the injection (from *t*_0_ = 0 h to *t*_1_ = 12 h) while the mice were still living was not considered. Therefore, the initially given dose (*D*_0_) from *t*_0_ = 0 h to *t*_2_ = 12 h was estimated from the observed dose (*D*) for *t*_1_ = 12 h to *t*_2_ = 43 h using the half-life decay curve as…

**FIGURE 4.**
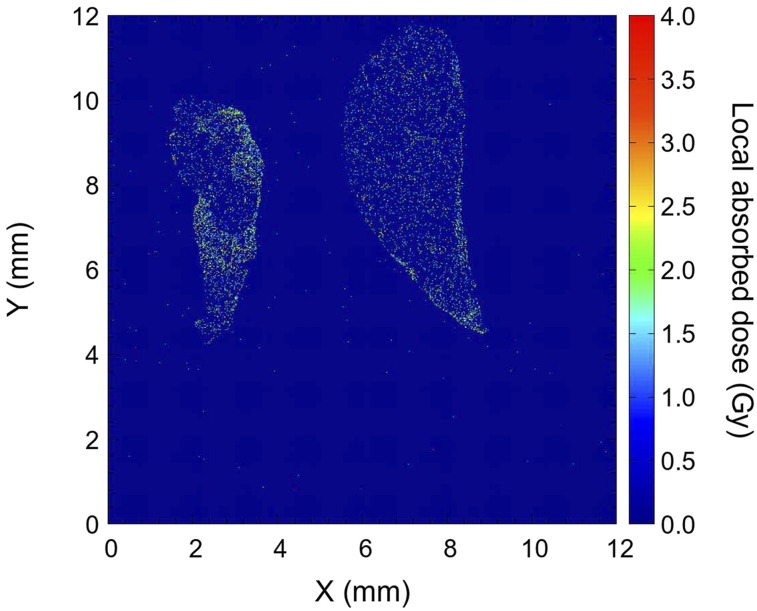
Contour map of total local absorbed dose in binned positions (Δ*x,* Δ*y*) with 10-μm intervals.

Eq. 4 $$\frac{D_{0}}{D}=\frac{\lambda }{\left(e^{-\lambda t_{1}}-e^{-\lambda t_{2}}\right)}\overset{t_{1}}{\underset{t_{0}}{\int }}e^{e^{-\lambda t}}dt,$$

where λ is a decay constant, that is, λ = ln(2)/half-life. Thus, estimated dose distributions of the initial dose (*D*_0_) for *t* = 0–12 h and the total dose for *t* = 0–43 h are shown as the blue and red histograms in Figure 3. In the total dose distribution, the given dose ranged from 1 to 7 Gy with a peak at around 2 Gy. The biologic equivalent dose is 2–3 times higher because the 5 MeV α-particle has a high relative biological effectiveness (29,30). The intensity map of the total dose is displayed in Figure 4, in which the deposited dose is significantly concentrated in the tumor region. A high-dose region is observed along the edge in the right-side tissue of Figure 4, where the tumors are specifically distributed. High-dose regions are also observed where the blood vessels are contained in the tissue. This estimation using Equation 4 is based on the assumption that the ^211^At-trastuzumab was immediately bound to the cancer cells after the injection at *t* = 0. We will soon investigate the possibility of varying the dose distribution in the tissues as a function of kinetic time in living mice.

## CONCLUSION

We investigated the concentration of ^211^At-labeled trastuzumab in liver metastases of mice by measuring α-particle tracks with the CR-39 plastic nuclear track detector. The concentration efficiency, the track density ratio of α-particle tracks in the necrotized tissue, where tumors were present, to the normal tissue, was found to be 6.0 ± 0.2. In the tumor region, the high-LET α-particles deposited a large enough dose to cause lethal damage to the cancer cells. The total absorbed dose ranged from 1 to 7 Gy with a peak at around 2 Gy, which would correspond to a 2–3 times higher biologically equivalent dose because of the high relative biological effectiveness of the α-particles emitted from ^211^At. The evidence of a significant concentration of α-particles in the tumors validated the effectiveness of ^211^At-trastuzumab for cancer radioimmunotherapy.

## DISCLOSURE

This work was partially supported by a Grant-in-Aid for Young Scientists (A) from the Japan Society for the Promotion of Science (JSPS KAKENHI grant 17H05093). No other potential conflict of interest relevant to this article was reported.
