# Supplementary material for: Evidence of Local Concentration of α-Particles from 211At-Labeled Antibodies in Liver Metastasis Tissue
Source: J Nucl Med. 2019 Apr;60(4):497–501. doi: 10.2967/jnumed.118.216853 (PMC6448461; doi:10.2967/jnumed.118.216853)
Supplement: Supplementary file 1 [file jnm216853SupplementalData.pdf]

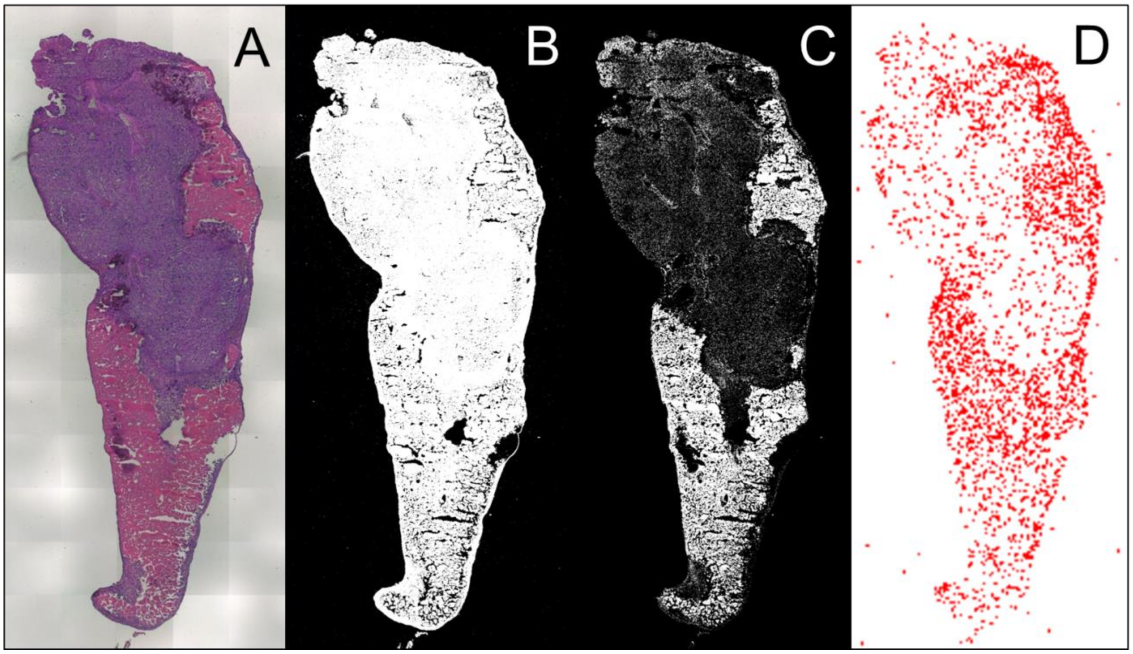

Supplemental Figure 1. A) The tissue extracted from Fig. 1A. B) The binarized image (in white) by setting a threshold level (138/256) in 8-bit grey scale for discriminating the background (in black). C) The binarized image for extracting the pink colour region (in white) by setting band-pass thresholds of 24-bit colour levels (red, 123-177; green, 42-84; blue, 94-139). D) The scatter plot of α-particle tracks on the tissue from Fig. 1B.
